# Private Things

**Published:** 1861-04

**Authors:** 


**PRIVATE THINGS.**—A person called some time ago, who in addition to a throat difficulty, complained that the urine had been coming away in a dribble for years, drop by drop, day and night. There was no remedy. No one can think of being in such a condition for a week without the most decided aversion, but to remain so, hopelessly, for all the long years of life yet to come and go in their weariness, is horrible to think of! The immediate cause of this distressing malady was a paralysis of the bladder, brought on by resisting the calls of nature to urination from early morning until business hours were over, and making it a habit day after day, on the ground that it interfered with business to give the requisite attention, and not knowing that any harm could come from it.

By retaining the urine too long, the bladder sometimes becomes so distended as to burst, and death is inevitable. When the membrane is not ruptured, it is, in a sense, like a bow bent to breaking, and loses all power of action; the urine can not be discharged; terrible pains ensue, and death is a speedy result. At other times persons get into the habit of resisting urination; this induces inflammation, reäbsortion into the circulation, and is a frequent cause of stone in the bladder, one of the most fearfully painful of human maladies, and when not fatal, requires a dangerous operation, at a cost of several hundred or a thousand dollars. This inability to urinate, brought on by deferring the calls, is, under all circumstances, a most distressing, dangerous, and alarming malady, and demands the most prompt and energetic treatment. The object of this article is not to propose a remedy, for but too often it proves fatal in two or three days; it is rather intended as a warning to all to avoid the cause by the easy means of yielding to nature's calls habitually and on the instant, however frequent. Medical books give a variety of fatal cases, where the patient was riding in a stage-coach, particularly in cold weather, and resisted nature for a whole day. Parents should teach their children that it is a false modesty and a false politeness to put off these calls under any circumstances whatever. It is a thing which should invariably be attended to the last thing at night, and the last thing previous to going to any public assembly, and as nothing can excuse an unnecessary risk of life, so nothing can excuse resistance to a call for urination.

While on the subject, it is well to state that the more a person exercises, the less will be the amount urinated, because the water of the system then passes through the pores of the skin. But when the weather is cold, these pores are to a certain extent closed; the water is then driven to the interior, and has to be passed off through the kidneys.

Ordinarily, the urine is high-colored and scant in warm weather, or when from exercise or other cause there is free perspiration; in cold weather it is abundant and clear. It is a practice hurtful and unwise to inspect the urine; its color, consistence, and quantity are modified by such a variety of circumstances of heat and cold, chill and fever, food and drink, and even by the emotions of the mind, that only a thoughtful physician can put a proper estimate on appearances, and even then, it must be in connection with all the facts of the case, bodily, mental, and moral.

Persons suffer a great deal in large cities from the want of public urinals. Scarcely a reader but may remember the time when he would have freely given a dollar for the use of such an institution. These establishments were formerly in Paris, but it was found impossible to keep them clean, and they were declared a nuisance. Hotels are scattered all through our cities, and while no proprietor of respectability would refuse an accomodation, yet if it could be brought about, that a tax of half a dime or a penny would secure it as a matter of bargain and sale, leaving both parties independent and free from obligation, much relief would be afforded and a great deal of suffering prevented. The whole subject merits the mature attention of every reader.

A very hasty and forcible attempt to urinate, especially when the parts are turgid, has resulted in a rupture of the membrane and subsequent stricture, and strictures tend to become more and more aggravated until urination can only be performed by introducing a tube into the bladder, the very thought of which, both as to the trouble and danger of it, well inspires dread. A patient once had practiced this for sixteen years, but on one occasion introducing the instrument carelessly, an artery was ruptured, causing death in a few hours. And yet not one reader in a hundred but thinks it a small matter, and without possible harm to resist the desire to urinate for hours together.

STOOLING.—By remaining too long at stool habitually, or by a sudden straining effort, with a view to expedition, the bowels have sometimes fallen down, at others, piles are engendered, as well as by the neglect to have one action of the bowels every twenty-four hours. Ailments of this sort aggravate themselves until it comes about that whenever the bowels act, their inner coating protrudes and the patient has to go to bed and remain there in literal agonies—" worse than death" is a common expression; sometimes these tortures last for two or three hours, to be repeated every day of the world, and yet between these sufferings the patient often appears in the enjoyment of perfect health. And how is such a terrible calamity induced? In one of three ways; remaining at stool over eight or ten minutes; straining rapidly; or third, by deferring the calls of nature until the body gets into the habit of calling every two or three days, instead of regularly every twenty-four hours, and that soon after breakfast. The practice of that

"Linked sweetness long drawn out,"

of which poets have sung, is competent to cause a life-long disablement. The lesson of the article is, a call of nature as to urination or stooling or the "delays" in the other regard, can never be resisted with impunity in any one single instance, and many a life has been embittered in consequence of ignorance of these things, a life which otherwise would have been one of sunshine and usefulness.

